# High-Fidelity MicroCT Reconstructions of Cardiac Devices Enable Patient-Specific Simulation for Structural Heart Interventions

**DOI:** 10.3390/jcm14207341

**Published:** 2025-10-17

**Authors:** Zhongkai Zhu, Yaojia Zhou, Yong Chen, Yong Peng, Mao Chen, Yuan Feng

**Affiliations:** 1 Department of Cardiology, West China Hospital, Sichuan University, Chengdu 610041, China; zhuzhongkai@wchscu.cn (Z.Z.); 2 Animal Experimental Center of West China Hospital, Sichuan University, Chengdu 610041, China; 3 Laboratory of Cardiac Structure and Function, Institute of Cardiovascular Diseases, West China Hospital, Sichuan University, Chengdu 610041, China; 4 Cardiac Structure and Function Research Key Laboratory of Sichuan Province, West China Hospital, Sichuan University, Chengdu 610041, China

**Keywords:** structural heart disease, micro-CT, reverse modeling, device simulation, preprocedural planning

## Abstract

**Background/Objective**: Precise preprocedural planning is essential for the safety and efficacy of structural heart interventions. Conventional imaging modalities, while informative, do not allow for direct and accurate visualization, limiting procedural predictability. We aimed to develop and validate a high-resolution micro-computed tomography (microCT)-based reverse modeling workflow that integrates digital reconstructions of metallic cardiac devices into patient imaging datasets, enabling accurate, patient-specific virtual simulation for procedural planning. **Methods**: Clinical-grade transcatheter heart valves, septal defect occluders, patent ductus arteriosus occluders, left atrial appendage closure devices, and coronary stents were scanned using microCT (36.9 μm resolution). Agreement was assessed by intra-class correlation coefficients (ICC) and Bland–Altman analyses. Device geometries were reconstructed into 3D stereolithography files and virtually implanted within multislice CT datasets using dedicated software. **Results**: Devices were successfully reverse-modeled with high geometric fidelity, showing negligible dimensional deviations from manufacturer specifications (mean ΔDistance range: −0.20 to +0.20 mm). Simulated measurements demonstrated excellent concordance with postprocedural imaging (ICC 0.90–0.96). The workflow accurately predicted clinically relevant parameters such as valve-to-coronary distances and implantation depths. Notably, preprocedural simulation identified a case at high risk of coronary obstruction, confirmed clinically and managed successfully. **Conclusions**: The microCT-based reverse modeling workflow offers a rapid, reproducible, and clinically relevant method for patient-specific simulation in structural heart interventions. By preserving anatomical fidelity and providing detailed device–tissue spatial visualization, this approach enhances preprocedural planning accuracy, risk stratification, and procedural safety. Its resource-efficient digital nature facilitates broad adoption and iterative simulation.

## 1. Introduction

Accurate preprocedural planning is critical to ensuring the safety and success of structural heart interventions [1,2,3]. Conventional imaging modalities, such as echocardiography and multislice computed tomography (MSCT), provide valuable anatomical information, but the prediction of device position and morphology after implantation still largely depends on operator experience and spatial imagination, which cannot be directly visualized. Although current imaging guidance combined with intraoperative adjustment achieves acceptable clinical outcomes, it is often associated with prolonged procedural times, increased rates of device repositioning, and elevated procedural risks. Therefore, direct visualization of the spatial relationship between interventional devices and surrounding anatomical structures is essential for optimizing preprocedural evaluation.

In recent years, three-dimensional (3D) printing of patient-specific cardiac structures has become an increasingly used adjunct for procedural simulation and preprocedural assessment in structural heart disease [4,5,6]. Despite its promising prospects, this technology faces practical limitations, including high costs, time-consuming production processes, and limited availability in most interventional centers [7,8,9,10,11]. Moreover, even 3D-printed models cannot accurately replicate the structural properties of tissues or the mechanical characteristics and dynamic deformation resulting from the interaction between tissues and interventional devices, particularly for cardiac nitinol implants. In contrast, Micro-computed tomography (microCT) has demonstrated exceptional spatial resolution [12,13,14,15] and enables the precise conversion of interventional devices into high-fidelity digital models. Importantly, pre-scanned device models can be stored and reused, supporting scalability and cost-effectiveness for routine clinical application. Integrating these device models directly into patient-specific imaging datasets preserves native anatomical detail while improving spatial visualization and facilitating a more intuitive understanding of device–tissue relationships for preprocedural planning.

To address existing limitations, we propose a microCT-based reverse modeling workflow that integrates the geometric configuration of metallic devices into original imaging datasets. This approach offers high spatial resolution, preserves anatomical fidelity, and enables efficient, patient-specific virtual simulation. By combining geometric accuracy with digital reusability, this method provides a practical and scalable alternative to current preprocedural planning techniques.

## 2. Materials and Methods

### 2.1. Study Design and Ethical Approval

This study was performed between July 2024 and April 2025. The study was approved by the institutional review board of West China Hospital (NO. 2021[1153]). The study complied with the Declaration of Helsinki and relevant national regulations regarding research ethics.

### 2.2. Device Samples

Devices included a transfemoral self-expanding transcatheter heart valve (THV), occluders for atrial and ventricular septal defects (A/VSD), a patent ductus arteriosus (PDA) occluder, a left atrial appendage (LAA) closure device, and a coronary stent. All devices were new, sterile, and unused clinical-grade implants obtained consecutively from the manufacturer’s test inventory for imaging evaluation.

### 2.3. Image Acquisition

Devices were scanned using a micro-computed tomography system (SkyScan 1276, Bruker, Kontich, Belgium). A suitable sample holder was selected according to device size. Scanning was performed at 70 kV tube voltage and 200 μA tube current with a 1 mm aluminum filter. Images were acquired at 1024 × 1024-pixel resolution, with a pixel size of 36.9 μm, using step-and-shoot mode at 0.02° rotation steps over 180°. Batch scanning mode was applied for long devices exceeding a single camera field of view. The average scanning time per device was approximately 9 min. Projection data were reconstructed using NRecon (Bruker) with histogram range set to 0–0.5, smoothing 0, ring-artifact reduction 6, beam-hardening correction 20 and automatic alignment correction; final volumetric image stacks were exported as DICOM files for downstream processing. For each device, a suitable sample holder was chosen according to device size and the device was oriented to minimize motion and to ensure full coverage of the imaging field; long devices were scanned in batch mode and subsequent stacks were stitched during reconstruction. All micro-CT scans were performed ex vivo on unused, clinical-grade implants. Once scanned, devices were reconstructed into SLA models and stored in a digital library for repeated use in patient-specific simulations, without the need for repeated scanning for each case. This ensures the feasibility of the workflow within routine preprocedural timelines and supports cost-effectiveness.

### 2.4. Image Processing and Reverse Modeling

**Figure 1:**
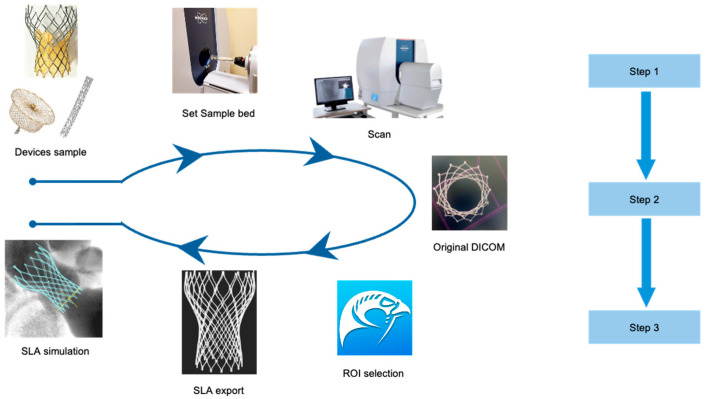
The process of 3D reverse remodeling and simulations. Step 1: Selection of a device size-matched sample bed and configuration of scan parameters for optimal imaging quality. Step 2: Acquisition of original DICOM data and initial preprocessing to ensure consistency and readiness for reconstruction. Step 3: Reconstruction of DICOM data into 3D SLA files for simulation purposes.

DICOMs were processed using Horos version 4.0.0 (Horos Project, Nimble Co. LLC, Annapolis, MD, USA). Absorption values were adjusted to optimize metal artifact suppression. Threshold segmentation was performed in Horos v4.0.0 using a metal-specific absorption threshold, initially set around 3000–4000 HU and then manually adjusted according to device type and image quality. ROI-growing was conducted in axial, coronal, and sagittal planes to ensure complete capture of device geometry, with manual correction when necessary. Absorption parameters were fine-tuned to minimize metallic artifacts while preserving the detailed morphology of the device frame. Regions of interest (ROI) for metal frames were defined using threshold segmentation, followed by ROI-growing in axial, coronal, and sagittal planes. Pixels outside the ROI were set to minimal values. Three-dimensional surface rendering was performed to reconstruct the device frame geometry. Processed models were exported as stereolithography (SLA) files for further measurements and simulation (Figure 1).

### 2.5. Preprocedural Simulations

SLA files were imported into FluoroCT software version 3.0 (Circle Cardiovascular Imaging) for virtual simulation within multislice CT datasets. The simulated spatial relationships between devices and anatomical structures were evaluated for procedural planning feasibility. Virtual implantation included alignment of device inflow/outflow axes, adjustment of implantation depth, and manual fine-tuning to match anticipated clinical deployment orientation. Standardized measurements (e.g., valve-to-coronary distances, implantation depth, disc-to-ostium distances) were taken directly within FluoroCT for subsequent comparison with postprocedural MSCT measurements.

### 2.6. Statistical Analysis

Agreement between simulated and post-procedural measurements was assessed using intra-class correlation coefficients (ICC) with two-way mixed effects models for absolute agreement. Bland–Altman analysis was conducted to calculate bias (mean difference) and 95% limits of agreement. Continuous variables were expressed as mean ± standard deviation. Relative differences between simulated and postprocedural measurements (ΔDistance, %) were calculated as the absolute difference divided by the postprocedural measurement, expressed as a percentage, and summarized as mean ± standard deviation. Analyses were conducted using SPSS version 26.0 (IBM Corp, Armonk, NY, USA).

## 3. Results

**Figure 2:**
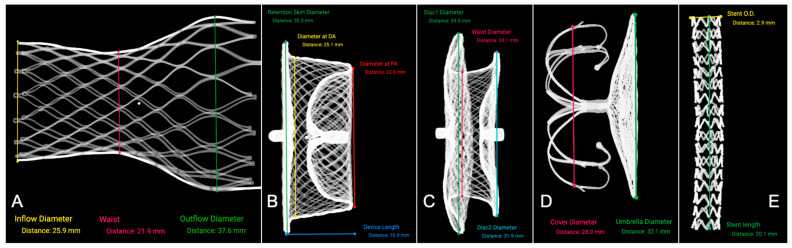
Representative 3D reconstructed SLA and corresponding measurements of the cardiac devices. (**A**) transcatheter heart valve (26 mm); (**B**) patent ductus arteriosus occluder (22 mm); (**C**) atrial septal defect occluder (24 mm); (**D**) left atrial appendage occluder (32 mm Disc); (**E**) Coronary stent (3.0 × 20 mm).

Between July 2024 and April 2025, six types of heart intervention devices were scanned for preprocedural evaluation, including transfemoral self-expanding heart valves (*n* = 8), ASD occluders (*n* = 4), VSD occluders (*n* = 2), LAA closure devices (*n* = 5), and PDA occluders (*n* = 3), coronary stent (*n* = 1). All devices were successfully scanned using microCT and reverse-modeled into 3D SLA files with a spatial resolution of 36.9 μm (Figure 2). These devices corresponded to 27 patients with THV implantation, 6 patients with ASD occluders, 3 patients with VSD occluders, 6 patients with LAA occluders, and 4 patients with PDA occluders. The baseline demographic and clinical characteristics of these patients (age, sex, diagnosis) are summarized in Supplementary Table S1.

Quantitative comparison between the reconstructed device dimensions and manufacturer nominal specifications demonstrated excellent accuracy. The mean ΔDistance values ranged from −0.20 mm to +0.20 mm, indicating negligible dimensional deviations across all device types. Full comparison details are provided in Table 1.

Quantitative comparisons between preprocedural simulated measurements and postprocedural MSCT measurements revealed consistently high agreement for all device types. The mean ΔDistance values ranged from 0.20 mm to 0.26 mm, with corresponding ICC between 0.90 and 0.96. Bland–Altman analyses demonstrated minimal biases and narrow 95% limits of agreement for each measured parameter (Table 2). These findings confirm the accuracy and clinical reliability of the microCT-based reverse modeling workflow.

### 3.1. Agreement Between Simulated and Postprocedural Measurements

#### 3.1.1. Transfemoral Self-Expanding Transcatheter Aortic Valve

**Figure 3:**
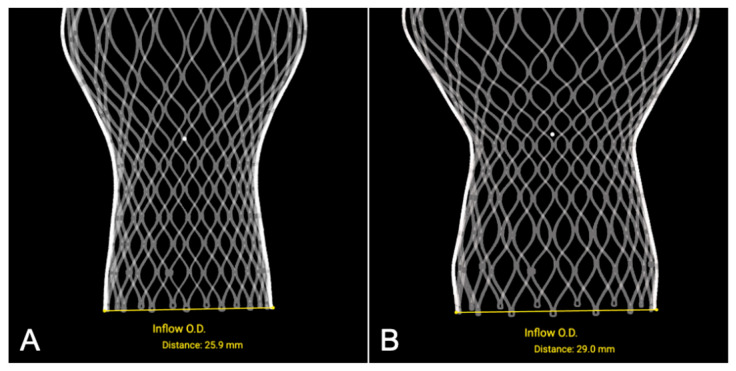
Measurements of reconstructed self-expanding valves: (**A**) 26 mm; (**B**) 29 mm. O.D.: outside diameter.

The reconstructed SLA models of the THV accurately preserved frame geometry and mechanical configuration, with the geometric parameters closely matching the nominal specifications of valve frames across all sizes and showing negligible dimensional deviation (Figure 3).

In eight patient-specific aortic root simulations, the mean simulated valve-to-left coronary distance was 3.16 ± 0.66 mm, compared with 3.40 ± 0.64 mm postprocedural (ΔDistance 0.24 ± 0.05 mm, ICC = 0.94). The valve-to-right coronary distance was 4.55 ± 0.70 mm vs. 4.77 ± 0.72 mm (ΔDistance 0.22 ± 0.05 mm, ICC = 0.92). For implantation depth, simulated mean values for the non-coronary cusp and left coronary cusp were 5.20 ± 0.30 mm and 6.36 ± 0.74 mm, respectively, compared with 5.40 ± 0.32 mm and 7.62 ± 0.73 mm post procedure.

**Figure A1:**
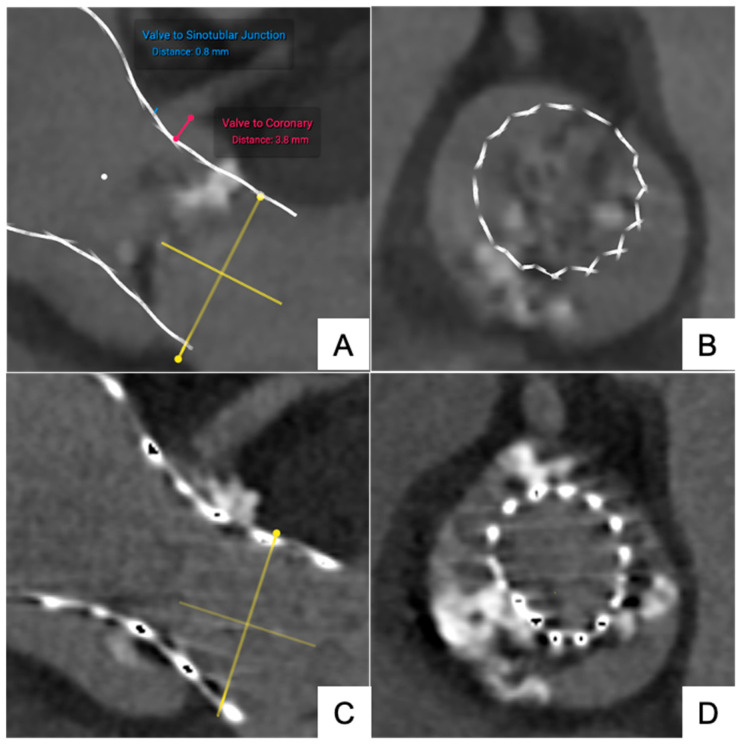
The preprocedural simulations and postprocedural CT images of TAVR. (**A**) Valve to coronary distance (red line) and valve to sinotublar junction (blue line) after virtual transcatheter heart valve insertion (THV) in long axis; (**B**) virtual THV insertion in short axis; (**C**,**D**) postprocedural CT images in long and short axis. Annular in yellow line.

Notably, one case of predicted coronary obstruction risk was confirmed postprocedural, validated by postprocedural elevated cardiac troponin T levels, and subsequent coronary angiography showing significantly reduced perfusion. Coronary flow was successfully restored after chimney stent placement (Figure A1).

#### 3.1.2. Occluders for ASD/VSD and PDA

High-resolution imaging clearly delineates the connectors, both discs, the central waist, and the metallic mesh frame, with no observed structural deformities.

**Figure 4:**
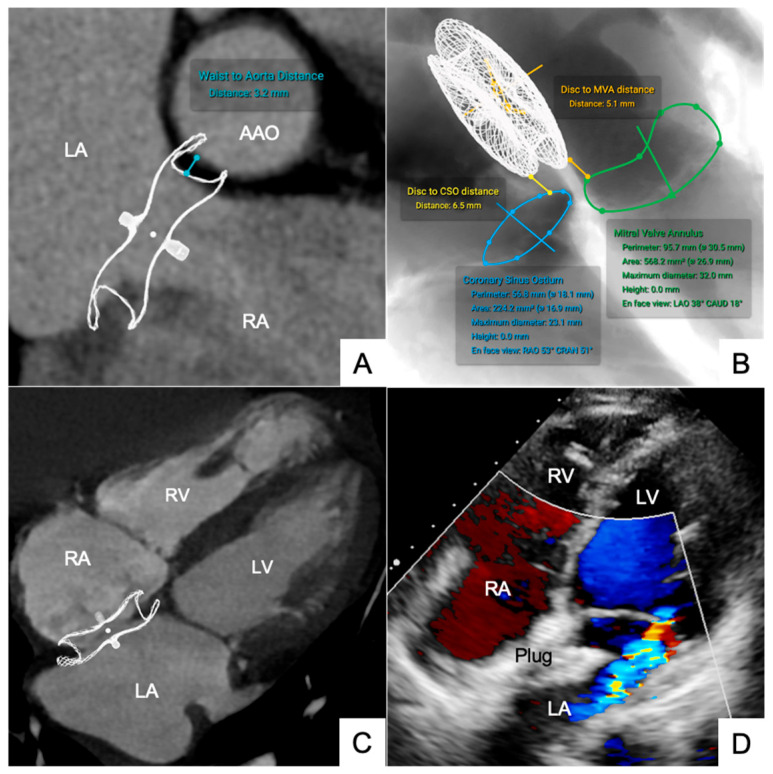
The measurement, simulation and result of atrial septal defect. (**A**,**B**) Preprocedural simulated measurement; (**C**) 24 mm occluder simulation in four chamber view; (**D**) postprocedural echocardiography.

**Figure A2:**
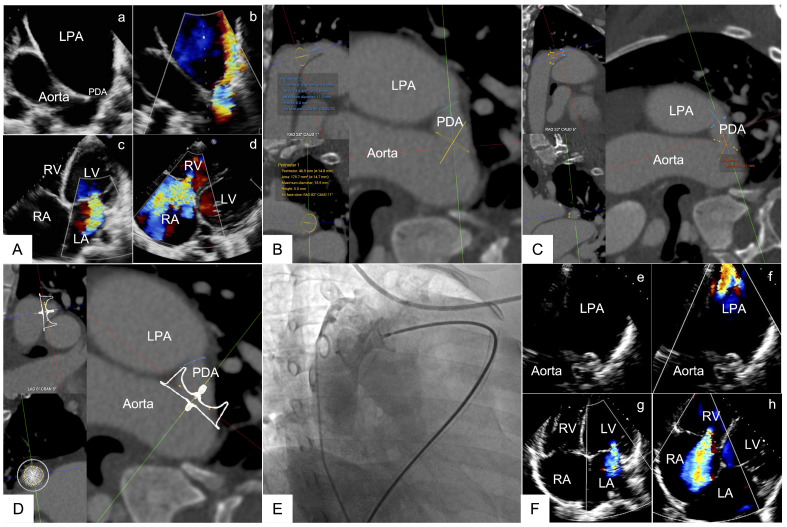
The measurement, simulation and result of patent ductus arteriosus (PDA). (**A**) preprocedural echocardiography: PDA without (a) and with (b) colored flow, sever mitral regurgitation (c), massive tricuspid regurgitation (d); (**B**,**C**): preprocedural measurements; (**D**) simulation of 24 mm PDA occluder; (**E**) PDA occluder deployment; (**F**) postprocedural echocardiography: occluded PDA without (e) and with (f) colored flow, moderate mitral regurgitation (g), severe tricuspid regurgitation (h). LPA: left pulmonary artery; RV: right ventricle; LV: left ventricle; RA: right atrium; LA: left atrium. Yellow arrowhead: plug.

The frame thickness ranges from 0.05 mm to 0.2 mm, aligning with manufacturer specifications. For ASD occluders, simulated waist-to-ascending aorta distance was 5.45 ± 2.40 mm versus 6.68 ± 2.42 mm postprocedural (ΔDistance 0.23 ± 0.04 mm, ICC = 0.95). The disc-to-mitral valve annulus (MVA) distance was 9.45 ± 2.40 mm simulated versus 10.68 ± 2.42 mm postprocedural (ΔDistance 0.23 ± 0.05 mm, ICC = 0.95) (Figure 4). For VSD occluders, the center-to-aortic valve annulus distance was 3.95 ± 1.48 mm simulated and 5.17 ± 0.51 mm postprocedural (ΔDistance 0.22 ± 0.06 mm, ICC = 0.91). These findings should be interpreted with caution given the very limited sample size (n = 3). For PDA occluders, simulated disc-to-aortic annulus margin distance was 4.05 ± 0.90 mm, compared with 3.97 ± 1.96 mm postprocedural (ΔDistance 0.24 ± 0.05 mm, ICC = 0.93) (Figure A2).

#### 3.1.3. LAA Occluder

Simulations accurately guided device selection and implantation positioning. Five LAA occluders were simulated.

**Figure 5:**
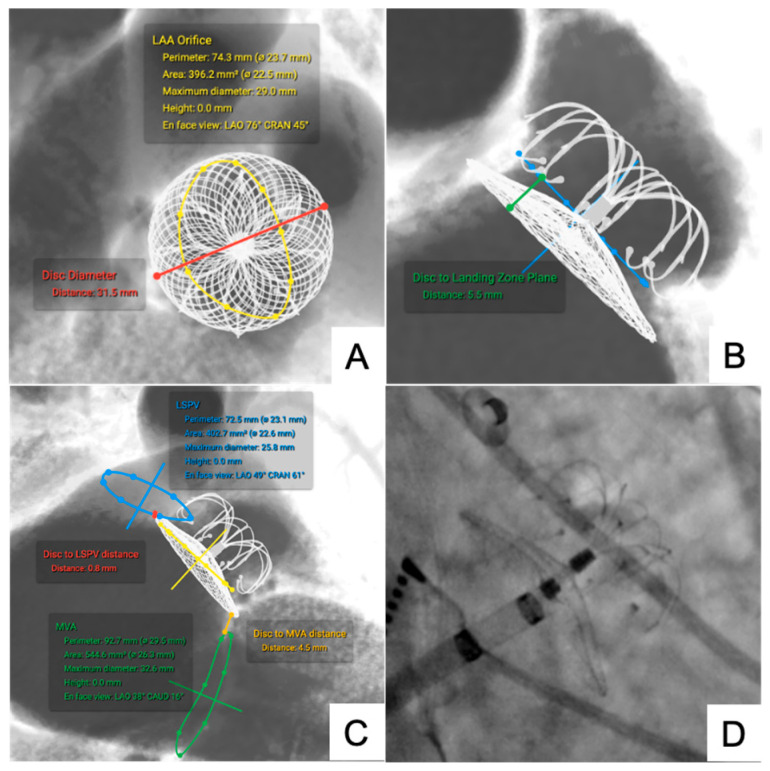
The measurement, simulation and deployment of left atrial appendage (LAA). (**A**–**C**) preprocedural simulated measurements; (**D**) LAA-2832 occluder deployment.

The disc-to-ostium distance was 6.12 ± 2.38 mm simulated versus 6.76 ± 2.42 mm postprocedural (ΔDistance 0.22 ± 0.06 mm, ICC = 0.93). The disc-to-left superior pulmonary vein (LSPV) distance was 3.80 ± 1.80 mm simulated versus 4.22 ± 2.34 mm postprocedural (ΔDistance 0.22 ± 0.05 mm, ICC = 0.91) (Figure 5).

All LAA closure procedures achieved successful device deployment without peridevice leaks or procedural complications.

## 4. Discussion

This study establishes a novel high-resolution micro-CT-based workflow, which is rapid, reproducible, and clinically relevant for structural heart interventions.

The present workflow benefits from pre-scanned device models, enabling rapid integration into patient-specific CT datasets without repeated micro-CT acquisition. This enhances scalability and cost-effectiveness, as device SLA models can be stored and reused for future procedures. Notably, the workflow successfully identified a high-risk case of coronary obstruction preprocedural, confirming its practical value for risk stratification in anatomically complex scenarios. Furthermore, integrating micro-CT-based reconstructions with advanced visualization techniques, such as holographic reality, could further improve procedural planning, risk stratification, and intra-procedural guidance [16].

Excellent agreement between simulated and postprocedural device measurements across multiple device categories (ICC 0.90–0.96) validates the translational accuracy and procedural relevance of this method. The observed absolute differences (ΔDistance, range 0.22–0.26 mm) between simulated and postprocedural measurements for THV were slightly larger than those between reconstructed models and manufacturer specifications (0.10–0.20 mm). This discrepancy is attributed to real device–tissue interactions such as frame deformation and compression during implantation, rather than inaccuracies in the geometric reconstruction. The consistently high ICC values (0.90–0.96) confirm the robustness of the workflow in predicting relative positions and trends despite small absolute differences. Future studies with larger, more diverse cohorts are warranted to confirm the broader applicability of this workflow across structural heart interventions.

Conventional imaging modalities such as echocardiography and MSCT remain indispensable in structural heart disease management. However, their ability to directly visualize the spatial relationship between interventional devices and patient-specific anatomy is limited, particularly in complex or borderline cases. Compared to conventional imaging and physical 3D printing, the proposed technique offers several compelling advantages. First, the sub-50 μm spatial resolution of micro-CT allows unparalleled delineation of fine device structures, connectors, and intricate geometrical configurations, overcoming the limitations inherent in multislice CT and echocardiography. Second, unlike computational finite element analyses or computational fluid dynamics models-which demand sophisticated software, meshing expertise, and boundary condition assumptions-this workflow employs accessible, widely available image processing tools for segmentation and 3D reconstruction. As such, it lowers the technical threshold for adoption, enabling routine procedural rehearsal, device sizing validation, and risk prediction in interventional centers with minimal computational infrastructure. Furthermore, the digitally reverse-modeled device files are reusable and scalable, supporting iterative simulations across multiple patient datasets, an attribute particularly valuable for complex structural interventions where multiple device configurations and implantation strategies may need evaluation.

Importantly, this workflow complements, rather than replaces, current imaging modalities. Although patient-specific 3D printing has emerged as a valuable adjunct for preprocedural simulation, its effectiveness is highly dependent on the quality of initial imaging data and it faces several limitations, including high production costs, time-intensive workflows, and limited material versatility [17,18]. Furthermore, 3D-printed anatomical models are typically single-use, leading to substantial material consumption and hindering scalability in clinical practice. These constraints emphasize the need for alternative solutions that combine precision, efficiency, and clinical practicality. Conversely, this digital simulation approach is resource-efficient, permits real-time virtual rehearsal, and provides a sustainable, scalable alternative that aligns with the operational demands of contemporary interventional cardiology and structural heart disease practice. Notably, by directly integrating real device geometries into native patient imaging, it offers a more anatomically faithful and intuitive simulation environment, facilitating improved spatial awareness and procedural planning without reliance on operator-derived spatial interpretation or invasive trial positioning.

Device simulations performed in this study yielded clinically relevant insights into device–anatomy interactions. For transfemoral self-expanding THV, simulations accurately reproduced device positioning, expansion morphology, and predicted critical anatomical relationships, including valve-to-coronary ostium distances and implantation depths. Notably, one case of predicted coronary obstruction risk was confirmed postprocedural, substantiating the clinical value of simulation-based risk assessment. Similarly, simulations involving ASD/VSD and PDA occluders demonstrated effective defect closure, secure device–tissue apposition, and appropriate relationships with adjacent cardiac structures [19]. The LAA closure device simulations confirmed optimal ostial coverage and anchoring depth, predicting procedural success without peridevice leaks or device dislodgment. These findings collectively underscore the importance of simulation-based preprocedural planning in reducing device-related complications and improving clinical outcomes.

This strategy also has strong implications for simulation-based training and operator credentialing. As structural interventions become increasingly complex and device portfolios expand, the capacity to perform repeated, anatomy-specific virtual simulations using true device geometries offers a powerful educational adjunct. Beyond individual procedural planning, this method provides a standardized platform for skill acquisition, procedural rehearsal, and device–anatomy interaction studies, potentially accelerating operator learning curves and promoting safer, more efficient procedural conduct.

The proposed workflow is limited by its reliance on SLA models that provide only geometric reconstructions and do not capture mechanical properties, deployment forces, tissue deformation, or paravalvular leak formation. Moreover, the small sample size in certain device subgroups restricts the generalizability of these quantitative results. Future studies involving larger and more diverse patient cohorts, combined with finite element analysis, are warranted to further validate the applicability of this workflow across a broader spectrum of structural heart interventions.

## 5. Conclusions

In conclusion, high-resolution microCT-based reconstruction enables precise characterization of structural heart devices and anatomy, providing a broadly applicable tool to support preprocedural planning and device selection in structural heart disease.

## Figures and Tables

**Table 1:** Comparison of Reconstructed Device Measurements with Manufacturer Nominal Specifications.

Device Type	Nominal Parameter (mm)	N	Reconstructed Parameter (mm)	ΔDistance (mm)
THV (Inflow)	23	3	22.9 ± 0.26	−0.10 ± 0.26
	26	3	25.9 ± 0.30	−0.10 ± 0.30
	29	2	29.1 ± 0.14	+0.10 ± 0.14
ASD Occluder (Left Disc)	18	1	17.8	−0.20
	20	2	20.1 ± 0.14	+0.10 ± 0.14
	24	1	23.9	−0.10
VSD Occluder (Left Disc)	10	1	10.1	+0.10
	12	1	11.8	−0.20
LAA Occluder (Umbrella)	28	2	28.1 ± 0.14	+0.10 ± 0.14
	32	2	31.8 ± 0.28	−0.20 ± 0.28
	34	1	33.9	−0.10
PDA Occluder (Left Disc)	8	1	7.9	−0.10
	10	1	10.1	+0.10
	22	1	12.2	+0.20
Coronary Stent	3.0 × 20.0	1	2.9 × 20.1	+0.01

Measurements are presented as mean ± SD. ΔDistance represents the mean difference between reconstructed and nominal measurements. THV = Transcatheter heart valve; ASD = Atrial septal defect; VSD = Ventricular septal defect; LAA = Left atrial appendage; PDA = Patent ductus arteriosus.

**Table 2:** Agreement Between Simulated and Postprocedural Measurements of Device–Anatomy Relationships.

Device Type	Measurement Parameter	Simulated (mm)	Postprocedural (mm)	ΔDistance (%)	ICC	Bias (mm)	95% LoA (mm)
THV (*n* = 27)	VTLC Distance	3.18 ± 0.58	3.42 ± 0.60	7.25 ± 1.84	0.94	0.24	−0.10 to +0.58
	VTRC Distance	4.57 ± 0.66	4.80 ± 0.68	2.42 ± 0.88	0.92	0.23	−0.15 to +0.61
	LCC Depth	6.34 ± 0.70	7.60 ± 0.72	4.05 ± 1.02	0.93	0.26	−0.13 to +0.65
	NCC Depth	5.22 ± 0.28	5.43 ± 0.30	3.81 ± 0.80	0.94	0.21	−0.09 to +0.51
ASD Occluder (*n* = 6)	Disc to MVA	9.48 ± 2.20	10.70 ± 2.32	3.15 ± 0.68	0.95	0.22	−0.10 to +0.54
	Disc to CSO	7.28 ± 2.82	9.05 ± 3.04	5.10 ± 1.16	0.96	0.21	−0.12 to +0.50
	Waist to AAO	5.48 ± 2.25	6.65 ± 2.30	3.12 ± 0.70	0.95	0.23	−0.11 to +0.56
LAA Occluder (*n* = 6)	Disc to MVA	6.10 ± 2.20	6.74 ± 2.30	3.88 ± 0.76	0.93	0.24	−0.11 to +0.59
	Disc to LSPV	3.82 ± 1.70	4.25 ± 2.20	2.60 ± 1.05	0.91	0.22	−0.10 to +0.55
	Disc to LZP	6.78 ± 2.05	7.20 ± 2.54	4.56 ± 1.64	0.92	0.21	−0.12 to +0.56
VSD Occluder (*n* = 3)	Center to AVA	3.96 ± 1.40	5.15 ± 0.48	3.70 ± 0.90	0.91	0.22	−0.10 to +0.53
	Center to AVN	13.78 ± 3.40	11.00 ± 4.20	12.50 ± 4.05	0.91	0.22	−0.11 to +0.54
PDA Occluder (*n* = 4)	Disc to AAM	4.06 ± 0.88	3.98 ± 1.90	2.45 ± 1.08	0.93	0.24	−0.13 to +0.62

Data are presented as mean ± SD. ΔDistance represents the absolute difference between simulated and postprocedural measurements. Intra-class correlation coefficients (ICC), mean bias, and 95% limits of agreement (LoA) from Bland–Altman analysis are reported for each parameter. THV = Transcatheter heart valve; VTLC = Valve-to-left coronary distance; VTRC = Valve-to-right coronary distance; NCC = Non-coronary cusp; LCC = Left coronary cusp; ASD = Atrial septal defect; MVA = Mitral valve annulus; CSO = Coronary sinus ostium; AAO = Ascending aorta; LAA = Left atrial appendage; LSPV = Left superior pulmonary vein; LZP = Landing zone plane; VSD = Ventricular septal defect; AVA = Aortic valve annulus; AVN = Atrioventricular node; PDA = Patent ductus arteriosus; AAM = Ascending aorta margin; ICC = Intra-class correlation coefficient; LoA = Limits of agreement.

## Data Availability

The original contributions presented in this study are included in the article/Supplementary Material. Further inquiries can be directed to the corresponding author(s).

## Supplementary Materials

The following supporting information can be downloaded at: https://www.mdpi.com/article/10.3390/jcm14207341/s1, Table S1: Baseline characteristics of the patient cohort.

## Abbreviations

The following abbreviations are used in this manuscript:

SLA	Stereolithography
CT	Computed tomography
DICOM	Digital imaging and communications in medicine
THV	Transcatheter heart valve
MSCT	Multi-slice computed tomography
microCT	Micro-computed tomography
ASD	Atrial septal defect
VSD	Ventricular septal defect
PDA	Patent ductus arteriosus
LAA	Left atrial appendage
ICC	Intra-class correlation coefficients
