# Supplementary material for: High-Fidelity MicroCT Reconstructions of Cardiac Devices Enable Patient-Specific Simulation for Structural Heart Interventions
Source: J Clin Med. 2025 Oct 17;14(20):7341. doi: 10.3390/jcm14207341 (PMC12565625; doi:10.3390/jcm14207341)
Supplement: Supplementary file 1 [file jcm-14-07341-s001.zip › jcm-3914066-supplementary.pdf]

Supplementary Table S1. Baseline characteristics of the patient cohort

Device Type	Number of Patients	Mean Age (years)	Age Range (years)	Male / Female (N)	Main Diagnosis	Device Type
THV	27	75.2	64–88	16 / 11	Severe aortic stenosis	THV
ASD Occluder	6	42.7	25–62	2 / 4	Atrial septal defect	ASD Occluder
VSD Occluder	3	34.3	18–50	2 / 1	Ventricular septal defect	VSD Occluder
LAA Occluder	6	73.1	67–82	4 / 2	Atrial fibrillation	LAA Occluder
PDA Occluder	4	29.5	12–45	3 / 1	Patent ductus arteriosus	PDA Occluder
